# Impact of acupuncture for allergic rhinitis on the activity of the hypothalamus-pituitary-adrenal axis: study protocol for a randomized controlled trial

**DOI:** 10.1186/s13063-019-3424-2

**Published:** 2019-06-20

**Authors:** Sheng Chen, Shu-Han Qu, Yi Zhang, Zhi-Hong Wen, Sheng-Nan Guo, Wei-Mei Zeng, Xue-Si Hou, Yi-Fan Jia, Yi Xiao, Federico Marmori, Jun Wang, Ji-Ping Zhao

**Affiliations:** 1 Department of Acupuncture and Moxibustion, Dongzhimen Hospital Affiliated to Beijing University of Chinese Medicine, Beijing, China; 2 Beijing Chaoyang Hospital, Beijing, China; 3 Institute of Acupuncture and Moxibustion, China Academy of Chinese Medical Sciences, Beijing, China; 4 European Foundation of TCM, Alicante, Spain; 5 Acupuncture Department, Medimar International Hospital, Alicante, Spain

**Keywords:** Allergic rhinitis, Hypothalamus-pituitary-adrenal axis, Randomized controlled trial

## Abstract

**Background:**

Patients with moderate and severe persistent allergic rhinitis (AR) have long-term physical and mental stress, leading to dysfunction of the hypothalamus-pituitary-adrenal (HPA) axis, which results in recurrence of AR. Previous research has proved acupuncture can regulate the function of the neuron-endocrine-immune system and contribute to improving the quality of life of patients with AR. This research aims to investigate the mechanism of acupuncture on the HPA axis in patients with moderate or severe persistent AR.

**Methods/design:**

This randomized controlled trial aims to study the impact of acupuncture on the HPA axis of patients with moderate and severe AR. This research also aims to compare the curative effects of different treatments in three groups of patients: those receiving western medicine, western medicine and conventional acupuncture, or western medicine and mind-regulating acupuncture. We will study the therapeutic effect of acupuncture and the correlation between the changes of therapeutic indexes and experimental indexes after the treatments. Therapeutic indexes include the Visual Analog Scale (VAS) of nasal symptoms and the Rhinoconjunctivitis Quality of Life Questionnaire (RQLQ) for AR patients; experimental indexes include corticotropin releasing hormone (CRH), adreno-corticotropic hormone (ACTH), cortisol (CORT), interleukin 4 (IL-4), and interferon-γ (IFN-γ).

**Discussion:**

The results of this trial will provide evidence for the influence of chronic, long-term, repeated stimulation in patients with moderate and severe persistent AR and the impact of acupuncture on the HPA axis of these patients.

**Trial registration:**

Acupuncture-Moxibustion Clinical Trial Registry, AMCTR-IOR-16000009. Registered on 22 August 2016.

## Background

The impact of allergic rhinitis (AR) is not limited to nasal symptoms. Based on previous studies, AR results in severe dyssomnia which causes a lack of energy during the day. This seriously affects the quality of life and work efficiency of AR patients. In adition, AR has negative impacts on the learning capability, attention, and behavior of children [1]. AR patients experience various unpleasant emotions and conditions, for example, fatigue, anxiety, tension, depression, and low sensibility [2]. A recent study about patients with persistent AR reported that 74% of 166 patients experienced psychological stress. Another study [3] found that the synthesis and secretion of cytokines such as interleukin (IL)-2, IL-6 and interferon (IFN)-γ are induced by psychological stress [4]. Psychological stress leads to imbalance of two groups of T helper cells, Th1 and Th2. The increase in Th2 causes the body to be more susceptible to allergens. Therefore, the initiation of AR includes both biological (allergen) and mental (stress, anxiety, and depression) factors.

At present, conventional medicine treatments can relieve the nasal symptoms of AR effectively. However, the psychology of AR patients is not being addressed by physicians. Attention to the psychological state of AR patients is insufficient and treatment regarding patients’ psychological states is not considered during regular treatment by most ear-nose-throat doctors. This limits the therapeutic effects of the treatments [5]. Clinical research found that the negative emotions of AR patients could be relieved if the acupoints responsible for the depression are relieved and tranquilization is used at the base of regular acupoints. As well as effective relief of the clinical symptoms, quality of life was clearly improved [6]. At the same time, due to the low toxicity and side effects, compliance with treatments increases. However, current research regarding the mechanism by which acupuncture regulates AR is limited. Besides, the index used in most research is mostly restricted to the inflammatory cells and cytokines. This results in a lack of a systemic and integral research approach.

In recent years, much research has proved that acupuncture can regulate the function of the neuroendocrine-immune (NEI) system [7, 8]. By stimulating the body’s “self-regulation system”, acupuncture enables the body to reach a dynamic balance and integration. Current research proves that the NEI system plays an important role in the pathological mechanisms of allergic diseases [9]. The NEI system of patients undergoes significant change under stress conditions. The main component of the NEI system is the hypothalamic-pituitary-adrenal (HPA) axis [10]. Under stress conditions, the body is affected by both physiological and mental factors. These changes can be long-term and excessive, leading to a series of physiological changes by interfering with the function of the neuroendocrine and immune systems. As a result, the Th1/Th2 balance is broken and the increase in Th2 induces a series of allergic diseases such as AR and asthma. This research aims to investigate the mechanism of action of acupuncture on the HPA axis in patients with moderate and severe persistent AR, and to explore the effect of acupuncture on the integral regulation of the immune system from different perspectives and multitargets. This will help to build a theoretical foundation for the therapeutic effect of acupuncture in various diseases.

## Methods

### Ethics and dissemination

Ethical approval for this trial has been granted by the Institutional Review Boards of Beijing University of Chinese Medicine (approval number BZYYYDX-LL-20150208), which is a central ethics committee providing approval for our two centers. Any adjustments to the scheme can only be carried out if the Ethics Committee and the volunteers give approvel after thorough communication. Written informed consent is required for all participants. The findings of the trial will be published in open access peer-reviewed journals.

### Trial design

**Fig. 1:**
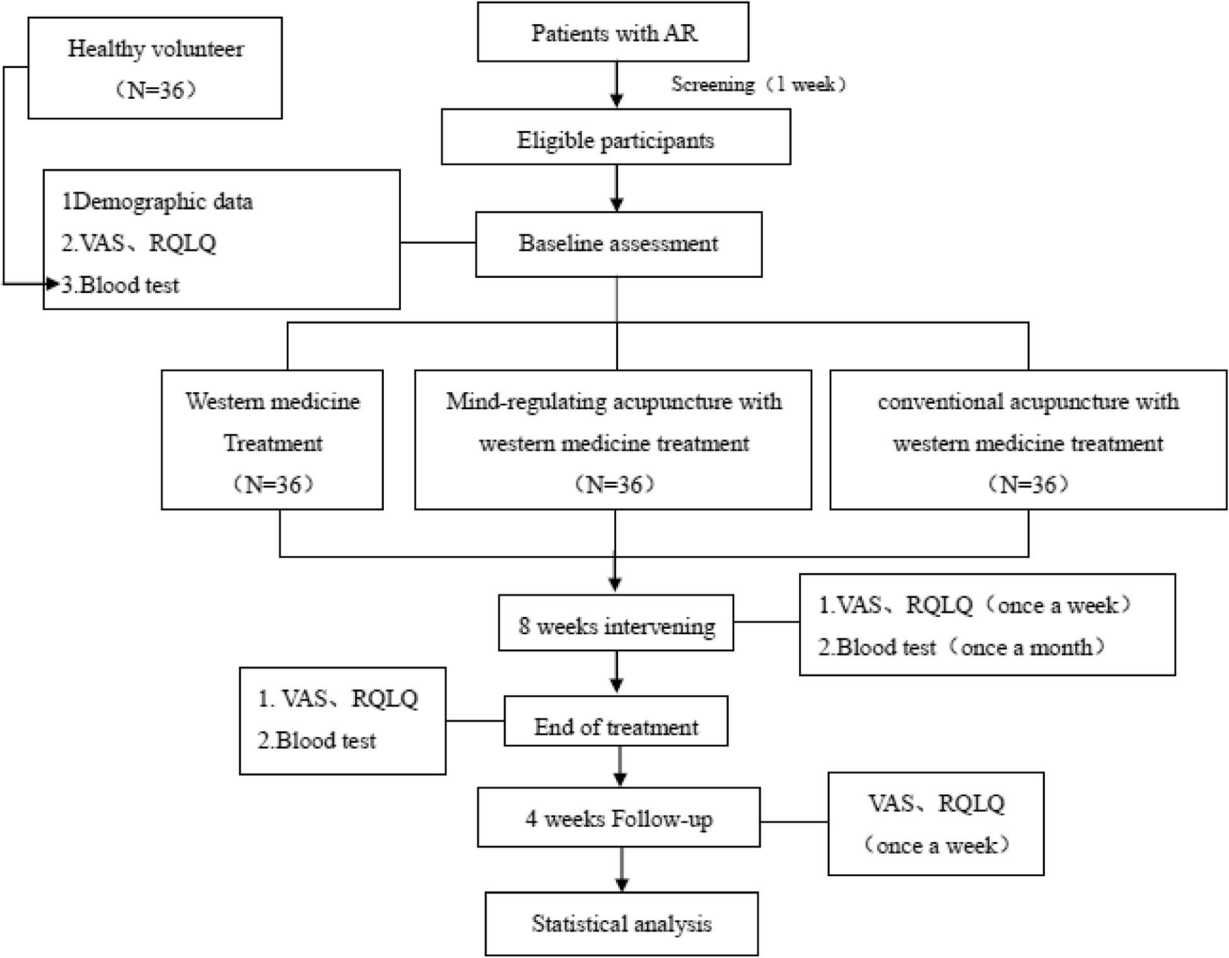
Study flow diagram. *AR* allergic rhinitis

**Fig. 2:**
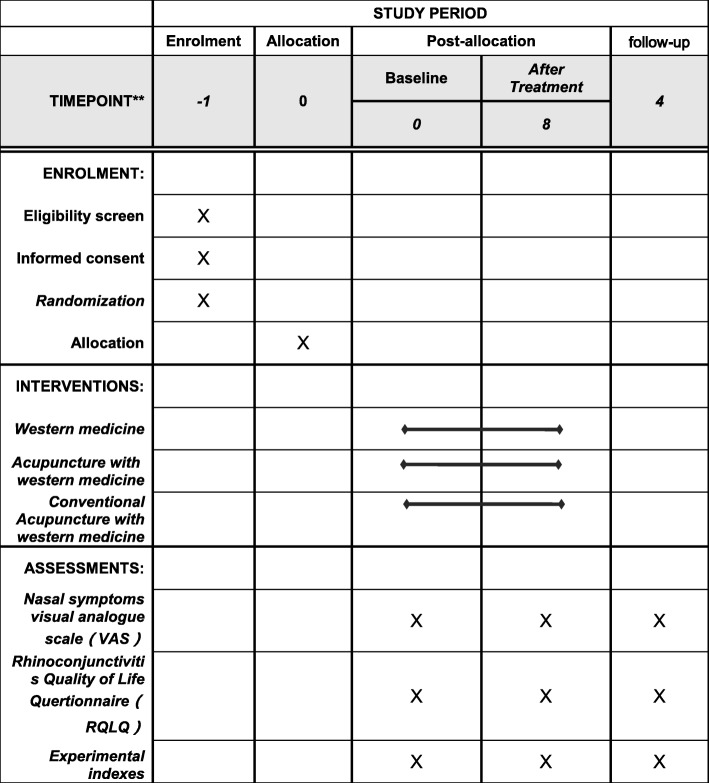
The schedule of enrolment, interventions, and assessments

This is a double-center, randomized controlled clinical trial of moderate and severe persistent AR. It has been registered in the Acupuncture-Moxibustion Clinical Trial Registry (AMCTR-IOR-16000009). This study will be carried out in Dongzhimen Hospital Affiliated to Beijing University of Chinese Medicine and Beijing Chao-Yang Hospital. The whole process will last for 13 weeks. After one week of assessment, participants will be randomly distributed into three groups to receive different treatments for 8 weeks, followed by a 4-week follow-up period. The three types of treatments are conventional acupuncture with western medicine treatment, mind-regulating acupuncture with western medicine treatment, and western medicine treatment (Fig. 1, 2).

### Participants

#### Inclusion criteria

Eligible participants should be diagnosed with moderate or severe persistent AR according to the criteria of Allergic Rhinitis and Its Impact on Asthma (ARIA) [11] and should reach the following requirements:

1. Persistent symptoms (> 4 days/week and > 4 weeks/year)
2. Aged 18–60 years
3. Mean Visual Analogue Scale (VAS) score for nasal symptoms ≥ 4 for 7 days during the screening period
4. Provide written, informed consent

#### Exclusion criteria

Individuals will be excluded from the study if they have one of the following criteria:

1. Individuals with acute respiratory infection, acute paranasal sinusitis, chronic paranasal sinusitis, organic lesions of the nasal cavity, or history of nasal surgery
2. Individuals with paroxysmal respiratory diseases such as asthma
3. Consumption of H1-antihistamines, steroids, antihistamine formulation, decongestant (applied to nasal cavity, oral cavity, or eyes), corticosteroids, antibiotics, and other medicines within 14 days; specific immunotherapy or systemic steroid treatment within 1 year
4. TCM physiotherapy or other traditional medicine such as acupuncture, moxibustion, cupping, and nasal inhalation within 14 days; consumption of Chinese medicine for AR within 14 days
5. Women who are pregnant, lactating, or undergoing preparation for pregnancy
6. Individuals with infectious diseases such as tuberculosis or hepatitis
7. Individuals who have smoked 10 cigarettes or more per day for 10 years or more
8. Individuals with scars at most selected acupoints or individuals who are uncooperative during the treatments

### Recruitment

Volunteers come through public notices and are AR patients of Dongzhimen Hospital Affiliated to Beijing University of Chinese Medicine, Beijing Chaoyang Hospital and community hospitals. After selections, volunteers who satisfy all requirements are required to sign the informed consent. These volunteers will undergo one week of assessment and they are required to fill in the VAS forms on nasal symptoms. After one week, volunteers with a mean Visual Analogue Scale (VAS) of nasal symptoms score of 4 or above for 7 days are selected for this clinical trial. They are required to examine the level of corticotropin releasing hormone (CRH), adreno-corticotropic hormone (ACTH), cortisol (CORT), IL-4, and IFN-γ in the blood. Volunteers who are not eligible are provided regular treatments for 4 weeks.

### Randomization and blinding

Using SAS 9.3.1 software (SAS Institute Inc., Cary, NC, USA), selected cases will be distributed in a 1:1:1 ratio to generate a randomized grouping table. Envelopes for the randomized grouping table will be prepared; the envelopes used are opaque, numbered in order, and contain the randomized serial numbers for grouping. According to the randomized serial numbers, individuals will be distributed to the observation groups and control group.

Researchers in charge of data collection, data statistics, and data evaluation will be blinded from the group allocation. However, individuals being treated and the physicians will not be blinded from the group allocation due to the different interventions in this trial.

### Planned interventions

#### Acupuncture treatment

Individuals from the observation groups will undergo either the acupuncture therapy or acupuncture with conventional treatment.

1. Acupoints: Bai Hui (DU20), Yin Tang (EX-HN3), Ying Xiang (LI20), Tai Chong (LR3), He Gu (LI4), Zu San Li (ST36); Da Zhui (DU14), Fei Shu (BL13), Pi Shu (BL20), Gan Shu (BL18), Shen Shu (BL23).
2. Needles: “Andy” disposable sterile stainless needles, size of 0.25 × 40 mm
3. Acupuncture methods: All acupoints are positioned according to the People’s Republic of China, State Standard Name and Location of Acupoints (GB 12346–2006) [12]. In the prone position, Da Zhui (DU14), Fei Shu (BL13), Pi Shu (BL20), Gan Shu (BL18), Shen Shu (BL23) are punctured. Once all acupoints achieve “deqi” (an irradiation feeling deemed to indicate effective needling), stimulation of the needles is manipulated in balancing moves (reinforcing-reducing method) for 30 s. Acupuncture treatments last for 20 min. In the supine position, Bai Hui (DU20), Yin Tang (EX-HN3), Ying Xiang (LI20), Tai Chong (LR3), He Gu (LI4), Zu San Li (ST36) are punctured. Once all acupoints achieve “deqi”, stimulation of the needles is manipulated in balancing moves (reinforcing-reducing method) for 30 s. Acupuncture treatments last for 20 min.

Mind-regulating acupuncture treatment is done with patients in both the prone and supine positions, whereas conventional acupuncture is done only in the supine position.

Acupuncture is carried out thrice a week and lasts for 8 weeks, for a total of 24 treatments in each group.

The western medicine treatment in the two acupuncture groups is the same as that in the western medicine group.

#### Western medicine treatment

Individuals in the control group will receive western medicine treatment.

According to the guidelines for the treatment of AR recommended by ARIA in 2008 [13], individuals in the control group will receive 2–4 weeks of intranasal corticosteroids, H1-antihistamines, or leukotriene receptor antagonists; after this, a specialized ENT examination will be repeated. An otolaryngologist will adjust the dosage or drug schedule according to the ENT findings, and treatments will be continued. One treatment course will last for 8 weeks.

### Outcomes

Therapeutic effect:

1. VAS of nasal symptoms score [14]

The scale includes overall nasal symptoms and differentiated nasal symptoms. The highest score for each section is 10, representing a symptom that is unbearable; the lowest score for each section is 0, representing a symptom with no influence. VAS score is accurate to one decimal place. It is used to evaluate the influence of overall nasal symptoms and differentiated nasal symptoms over the past week.

Data will be collected before the start of treatment (the baseline assessment) and at the end of each week during the treatment and follow-up periods.

2. Rhinoconjunctivitis Quality of Life Questionnaire score [15]

The Rhinoconjunctivitis Quality of Life Questionnaire (RQLQ) includes 24 questions in seven sections: activity, sleeping, symptoms unrelated to nose and eyes, actual problems, nasal symptoms, ocular symptoms, and emotion. The score for each question is divided into seven levels: 0 = no influence, 1 = almost no influence, 2 = some influence, 3 = moderate influence, 4 = bad influence, 5 = worse influence, 6 = worst influence. The score is calculated separately in each section and the total score of these seven sections is considered as the RQLQ score. The highest possible score is 144. The scale is used to evaluate the influence of rhinitis symptoms on patient quality of life.

Data will be collected before the start of treatment (the baseline assessment) and at the end of each week during the treatment and follow-up periods.

The experimental indexes are serum CRH, plasma ACTH, cortisol, serum IL-4, and IFN-γ.

Data will be collected before the start of treatment (the baseline assessment), at the end of treatment, and 4 weeks after the treatment.

### Adverse events

Information on adverse events will mainly depend on the conscious feedback of individuals. Prior guidance is necessary for all volunteers. If an adverse event occurs, physicians will check the patients’ condition immediately and decide if any necessary examinations and treatments will be carried out. For serious adverse events, the trial will be interrupted immediately and proper steps will be taken, and detailed information on incidents will be recorded at the same time.

### Sample size

This study is exploratory research designed to investigate the mechanism of acupuncture treatment for AR patients. With reference to the first stage of the clinical trial in the national drug register published in 2005, also based on a preliminary study and other research findings, the required sample size is 90; taking into account a 20% drop-out rate, a total of 108 cases will be recruited. Dongzhimen Hospital and Beijing Chaoyang Hospital are each responsible for 50% of recruitment for the clinical study. Another 36 healthy volunteers will be recruited as the control group, for a total of 144 cases.

### Statistical analysis

Data from this clinical trial will be analyzed using SPSS version 21.0 software (released 2012, IBM Corp., Armonk, NY, USA). Demographic (age, sex, course, family history, et al.) and clinical data will be used as the baseline assessment to compare the two groups. The main and second indexes of therapeutic effects will be measured as mean ± standard deviation. Differences before and after the treatment in each group will be compared by paired *t*-test. ANOVA will be used in the comparison between groups. Count data will be measured by constituent ratio while chi-square test or non-parametric test will be used in the comparison between groups. *P*<0.05 indicates the difference is statistically significant.

## Discussion

AR has become a global and intractable disease. Besides negative impacts on patients’ quality of life, it results in various kinds of diseases which may cause the loss of working ability and life. Although the symptoms can be relieved effectively by various topical or oral drugs, susceptibility to the disease cannot be changed. Specific immunotherapy can relieve the allergic symptoms, but long-term treatment always results in poor compliance [16]. Acupuncture, on behalf of traditional treatments, reflects the emphasis of TCM on “mind-regulation”. In all kinds of diseases, especially physical and mental disorders, treatments which emphasize both a patient’s physiological and mental state, play an important role. Recent research proves that [17] AR is a physical and mental disorder, being closely related to social psychological factors. AR patients, especially those with moderate and severe disease, are likely to have mental and behavior problems due to the chronic and unstable symptoms, continuous and unsuccessful treatments, accumulated medical expenses, and effects on their social function. Furthermore, a patient’s mental and behavior problems may aggravate their illness [18]. As a result, a vicious cycle is formed.

In 2001, WHO organized a discussion, attended by Otolaryngology experts from around the world, on the impact of AR on asthma and the concept of “one airway, one disease” has been proposed. Except for differences in their clinical symptoms, AR and asthma are similar in various aspects, such as pathogenic and pathological changes [19]. At present, the HPA axis has become the focus in research on the mechanisms involved in AS [20]. Some scholars feel that the HPA axis of AR patients is likely to be activated. After stimulation of patients’ nasal cavities by allergens, the detection of ACTH and cortisol in the peripheral blood circulation indicates that AR can activate the HPA axis [21]. However, by considering the complexity of the pathogenic mechanism of AR, the influence of chronic, long-term, repeated stimulation of the HPA axis of patients with moderate or severe persistent AR is unclear.

On the basis of a large number of references and related research, a hypothesis is suggested that patients with moderate or severe persistent AR undergo a long-term change in stress status both physically and mentally. High stimulation activates the HPA axis continuously, with excessive secretion of hormones resulting in selective inhibition of Th1 cells. The balance tends to move towards the humoral immune system mediated by Th2 and this increases patients’ susceptibility. On the other hand, long-term activation of the HPA axis may induce HPA axis exhaustion and lead to its dysfunction, resulting in lost control and limitation of the allergic inflammation. This may be one of the reasons for AR recurrence. Acupuncture can relieve patients’ nasal symptoms and modulate their psychological state by regulating hormones and cytokines of the HPA axis. This moderates the functions of the NEI system to relieve the allergic inflammation of AR. However, this RCT is an exploratory study with limited experience. Therefore, the sample size, outcome evaluation items, and follow-up pattern should be improved in the future.

## Trial status

Ongoing.

## Data Availability

On completion, anonymized data obtained in the trial will be available from the corresponding author on reasonable request.

## References

[1] Craig TJ, Sherkat A, Safaee S (2010). Congestion and sleep impairment in allergic rhinitis. Curr Allergy Asthma Rep.

[2] Chen S, Guo SN, Zhang Y, Nan YN, Zhao JP (2014). Research progress on the relationship between physical symptoms and psychological disorders in patients with allergic rhinitis. J Emerg Tradit Chin Med.

[3] El HDD, Ahmed MR, Farid AM. Psychological stress and its relationship with persistent allergic rhinitis. European Archives of Oto-Rhino-Laryngology. 2016;273(4):899–904. 10.1007/s00405-015-3641-6 25951791

[4] Wang HX, Yang SM (2015). Can not ignore the allergic rhinitis patients with mental and behavioral disorders. Natl Med J Chin.

[5] Xi L, Han DM, Lv XF, Zhang L (2009). Psychological characteristics in patients with allergic rhinitis and its associated factors analysis. Chin J Otorhinolaryngology Head Neck Surg.

[6] Chen S, Wang J, Bai P, Zhao Q, Tan C, Wang BK (2015). Moderate and severe persistent allergic rhinitis treated with acupuncture: a randomized controlled trial. Chin Acupunct Moxibustion.

[7] Ding S. S., Hong S. H., Wang C., Guo Y., Wang Z. K., Xu Y. (2013). Acupuncture modulates the neuro-endocrine-immune network. QJM.

[8] Chen B, Ming-Yue LI, Dind SS (2014). Research progress on regulations on nerve-endocrine-immune network by acupuncture. World J Acupunct Moxibustion.

[9] Marshall GD, Agarwal SK (2000). Stress, immune regulation, and immunity: applications for asthma. Allergy Asthma Proc.

[10] Faravelli C, Lo SC, Lelli L (2012). The role of life events and HPA axis in anxiety disorders: a review. Curr Pharm Des.

[11] Brożek JL, Bousquet J, Baena-Cagnani CE (2010). Allergic Rhinitis and its Impact on Asthma (ARIA) guidelines: 2010 revision. J Allergy Clin Immunol.

[12] General Administration of Quality Supervision, Inspection and Quarantine of the People's Republic of China, Standardization administration of the People's Republic of China. People's Republic of China the state standard the name and location of acupoints (GB 12346–2006). Beijing: Standards Press of China. 2006;8–34.

[13] Bousquet J, Khaltaev N, Cruz AA (2008). Allergic Rhinitis and its Impact on Asthma (ARIA) 2008. Allergy.

[14] Bousquet PJ, Combescure C, Neukirch F (2007). Visual analog scales can assess the severity of rhinitis graded according to ARIA guidelines. Allergy.

[15] Juniper EF, Guyatt GH (1991). Development and testing of a new measure of health status for clinical trials in rhinoconjunctivitis. Clin Exp Allergy.

[16] Valovirta E, Ryan D (2008). Patient adherence to allergic rhinitis treatment: results from patient surveys. Medscape J Med.

[17] Chida Y, Hamer M, Steptoe A (2008). A bidirectional relationship between psychosocial factors and atopic disorders: a systematic review and meta-analysis. Psychosom Med.

[18] Montoro J, Mullol J, Jáuregui I (2009). Stress and allergy. J Investig Allergol Clin Immunol.

[19] Xu WG, Li MH (2014). The relationship between allergic rhinitis and asthma. Chin J Clin.

[20] Deng M, Zhang WN (2014). Clinical research of Gubendingchuan decoction intervening NEI net unbalance of patients with bronchial asthma. J Emerg Tradit Chin Med.

[21] Kalogeromitros D, Syrigou EK, Makris M (2007). Nasal provocation of patients with allergic rhinitis and the hypothalamic-pituitary-adrenal axis. Ann Allergy Asthma Immunol.

